# Autochthonous Chikungunya Fever in Traveler Returning to Japan from Cuba

**DOI:** 10.3201/eid2209.160603

**Published:** 2016-09

**Authors:** Motoyuki Tsuboi, Satoshi Kutsuna, Yasuyuki Kato, Eri Nakayama, Ken-ichi Shibasaki, Shigeru Tajima, Tomohiko Takasaki, Yuichi Katanami, Kei Yamamoto, Nozomi Takeshita, Kayoko Hayakawa, Shuzo Kanagawa, Norio Ohmagari

**Affiliations:** National Center for Global Health and Medicine, Tokyo, Japan (M. Tsuboi, S. Kutsuna, Y. Kato, Y. Katanami, K. Yamamoto, N. Takeshita, K. Hayakawa, S. Kanagawa, N. Ohmagari);; National Institute of Infectious Diseases, Tokyo (E. Nakayama, K.-i. Shibasaki, S. Tajima, T. Takasaki)

**Keywords:** chikungunya fever, chikungunya virus, travel, Cuba, conjunctivitis, imported viral diseases, viral infections, viruses, dengue, Zika virus, phylogeography, Japan, global health

**To the Editor:** Chikungunya fever is a febrile illness caused by mosquito-transmitted chikungunya virus CHIKV: (genus *Alphavirus*, family *Togaviridae*). Clinical signs and symptoms typically begin with high-grade fever after an incubation period of 2–4 days (*1*). Other common symptoms include polyarthralgia, which is usually symmetric and involves multiple and distal joints, and skin involvement manifesting as a macular or maculopapular rash (*2*). Peripheral lymphadenopathy (most often cervical) and conjunctivitis might also occur (*3*).

Since late 2013, several outbreaks of illness caused by CHIKV have occurred in the Americas, including South America, the Caribbean, and the United States, which are outside this virus’s former distribution area (*3*). Although autochthonous transmission of chikungunya fever has been reported in most Caribbean islands, only imported cases have been previously reported in Cuba (*4*). As increased numbers of US tourists visit Cuba after improved diplomatic relations in July 2015, reports of chikungunya fever cases in Cuba are of interest for travelers and healthcare providers. We describe a case of autochthonous chikungunya fever in a man who had traveled from Japan to Cuba.

In late February 2016, a previously healthy 27-year-old man visited a travel clinic in the National Center for Global Health and Medicine (Tokyo, Japan) with fever and rash. In mid-February, he had traveled to Havana and Santiago de Cuba in Cuba by way of Toronto, Ontario, Canada, for 11 days of sightseeing. He used no insect repellent during the trip and was unaware of any mosquito bites. When he sought care, he reported a high-grade fever (39°C) for 24 hours and several symptoms since the day of his return: retro-orbital pain, malaise, congested conjunctivas, and a rash on his anterior chest. Over the previous few days, his knee and ankle joints also had mild arthralgia.

On physical examination, the patient’s body temperature was 38.7°C, and he had congested bulbar conjunctivas, cervical lymphadenopathy, and maculopapular rashes on his face, trunk, and extremities (Technical Appendix, Figure, panels A, B). Laboratory tests revealed lymphopenia (701 cells/μL) and mild elevation of C-reactive protein (0.87 mg/dL). Real-time reverse transcription PCR detected CHIKV RNA in his serum sample. Phylogenetic analysis was performed on the basis of nucleotide sequences of the E1 gene from the sample by using the maximum likelihood method with 1,000 bootstrap replicates and MEGA 6.0 software (*5*). This sequence (GenBank accession no. LC146714) was 99.9% (1,319 of 1,320 sequences) was identical to that of a CHIKV strain isolated from the Dominican Republic in 2014 (GenBank accession no. KR559498) (Figure; Technical Appendix Table). The positive-to-negative ratio of CHIKV-specific IgM was negative in a serum sample collected on day 4 after fever onset but was positive in a sample taken 7 days later (positive-to-negative ratios 5.6 and 21.9, respectively; ratios were considered positive if >11). Because the patient’s serum samples contained no dengue or Zika virus, infections from these viruses were excluded, and chikungunya fever was diagnosed.

**Figure:**
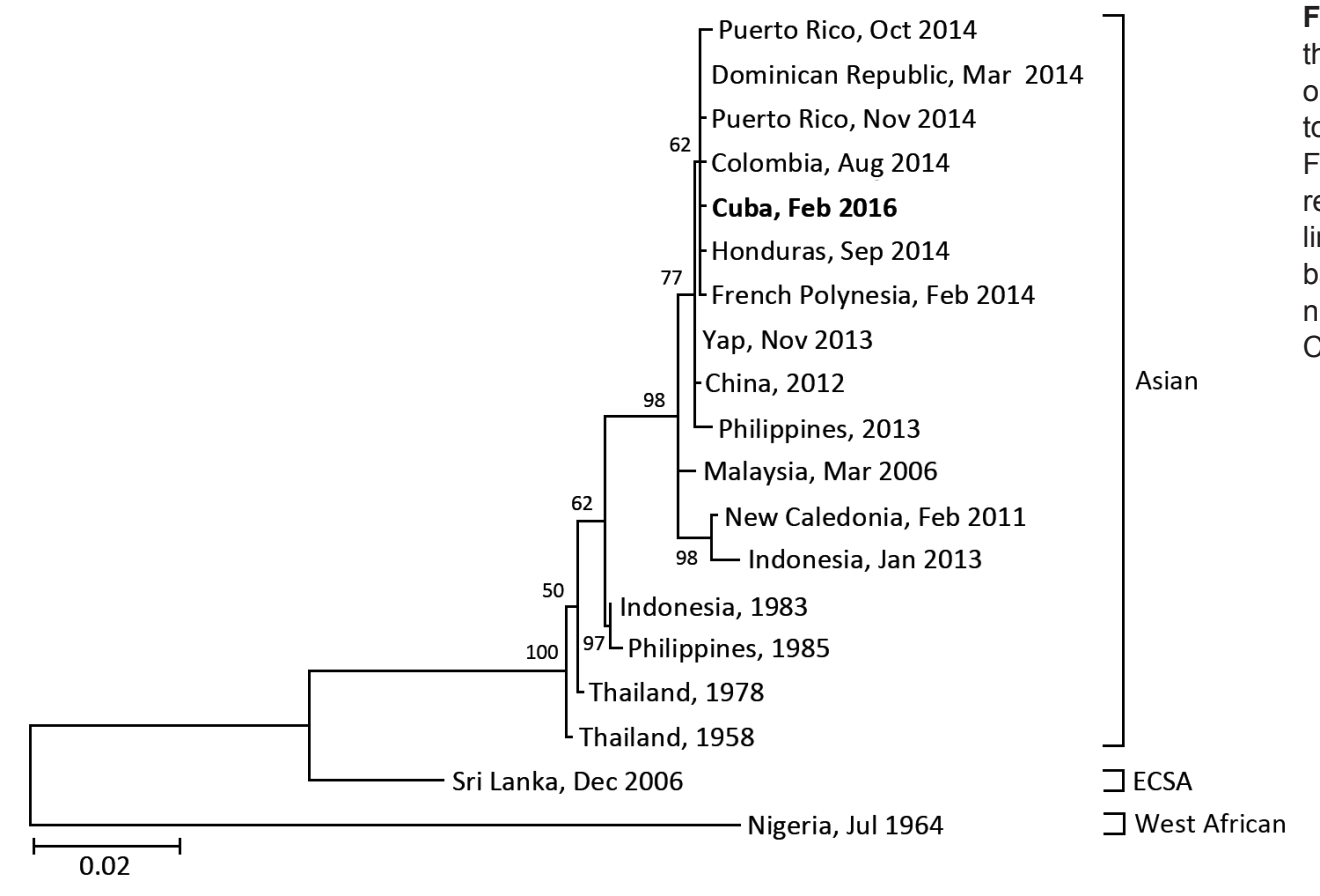
Phylogenetic analysis of the chikungunya virus sequence obtained from a patient returning to Japan (in bold) from Cuba in February 2016, compared with reference sequences. Virus lineages are shown at right. Scale bar represents substitutions per nucleotide position. ECSA, Eastern/Central/South African lineage.

One day after the patient’s first visit to the clinic, rashes on his extremities became worse and slightly itchy. Pain also developed in his wrists and metacarpophalangeal joints of his hand, followed by cervical pain and slight rigidity on the hand’s distal and proximal interphalangeal joints. The patient was initially treated with acetaminophen (600 mg 3×/d 2 d); after diagnosis of chikungunya fever, he was treated with loxoprofen and rebamipide (60 mg and 100 mg, respectively, 3×/d 7 d). The congested bulbar conjunctivas and rash on his trunk improved; soon thereafter, all symptoms resolved.

CHIKV was first isolated in 1953 in Tanzania during an epidemic outbreak in East Africa (*6*). Mosquitoes, predominantly *Aedes aegypti* and *Ae. albopictus*, transmit the virus (*2*). *Aedes* spp. are also the common vector of dengue and Zika viruses, and localized dengue outbreaks occurred in Santiago de Cuba in 1997 and in Havana in 2000–2001 because of the persistence of *Aedes* mosquito infestation in Cuba (*7**,**8*). Furthermore, autochthonous Zika virus infection in Cuba was first reported in March 2016 (*9*).

Differentiation between chikungunya fever, dengue fever, and Zika virus infection is difficult because of similar signs and symptoms and common endemic areas. We suspected chikungunya fever in this patient because of high-grade fever and maculopapular rash, although he also had prominent conjunctivitis, which is uncommon in CHIKV-infected patients but frequent in persons infected with Zika virus (*3**,**10*). Phylogenetic analysis of the virus isolated from this patient revealed a high sequence homology with recent strains discovered in Caribbean and Central American countries in 2014. Homology between the isolate from this patient and a 2014 Asian lineage isolate from the Dominican Republic was 99.92% at the nucleotide level. 

This case highlights the potential threat of a chikungunya fever outbreak in Cuba. Physicians should consider chikungunya fever in the differential diagnosis for febrile travelers returning from Cuba with a rash, similarly to patients returning from other countries in which dengue fever, chikungunya fever, and Zika virus infection are endemic. Preventive measures, including advice to travelers on proper use of insect repellents, are critical for preventing CHIKV infection.

Technical Appendix Congested bulbar conjunctivas and maculopapular rash on trunk of patient returning to Japan from Cuba and chikungunya viral strains used for phylogenetic analysis.
