# Supplementary material for: Autochthonous Chikungunya Fever in Traveler Returning to Japan from Cuba
Source: Emerg Infect Dis. 2016 Sep;22(9):1683–5. doi: 10.3201/eid2209.160603 (PMC4994370; doi:10.3201/eid2209.160603)
Supplement: Technical Appendix — Congested bulbar conjunctivas and maculopapular rash on trunk of patient returning to Japan from Cuba and chikungunya viral strains used for phylogenetic analysis. [file 16-0603-Techapp-s1.pdf]

Article DOI: http://dx.doi.org/10.3201/eid2209.160603

Autochthonous Chikungunya Fever in Traveler from Japan to Cuba

Technical Appendix

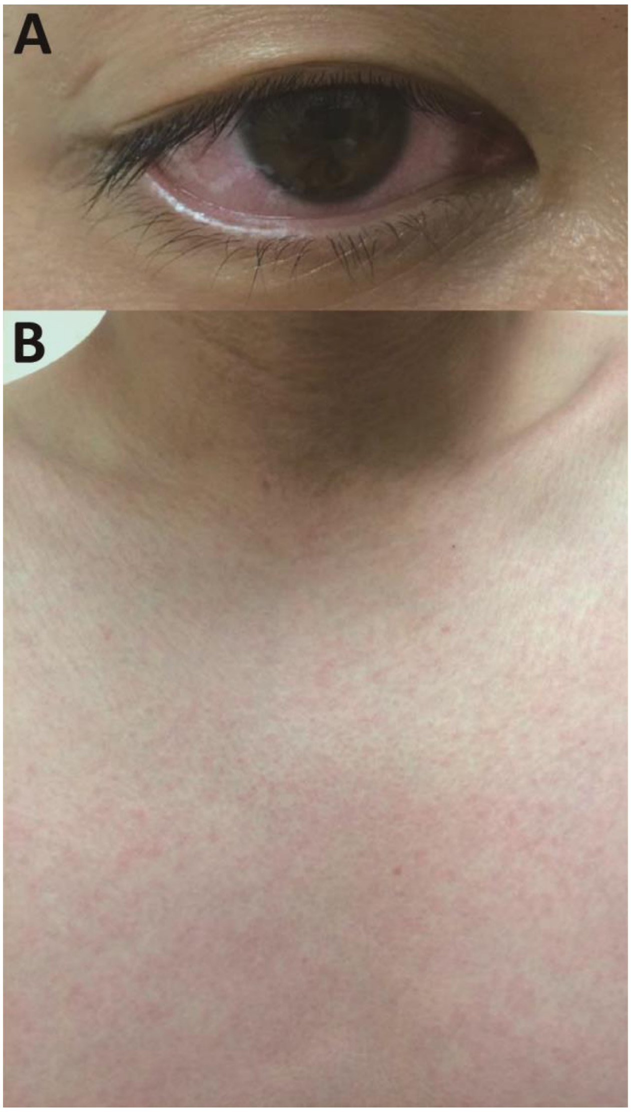

Technical Appendix Figure. Congested bulbar conjunctivas and maculopapular rash on patient's trunk. A) Congestion of bulbar conjunctivas resolved after a few days. B) Maculopapular rash with pruritus spread from the trunk to the extremities.

Technical Appendix Table. Chikungunya viral strains used for phylogenetic analysis*

Lineage	Accession no.	Strain name	Country	Date
Asian	KR559470	WHCHK1	Puerto Rico	Nov 2014
	KR559473	WHCHK4	French Polynesia	Feb 2015
	KR559483	WHCHK14	Puerto Rico	Oct 2014
	KR559488	WHCHK19	Honduras	Sep 2014
	KR559491	WHCHK22	Colombia	Aug-2014
	KR559498	WHCHK29	Dominican Republic	Mar 2014
	HE806461	NC/2011-568	New Caledonia	Feb 2011
	KM673291	DH130003	Indonesia: Bali	Jan 2013
	FN295483	MY/06/37348	Malaysia: Perak	Mar 2006
	KC488650	CHIKV-JC2012	China	2012
	KJ689453	Yap 13-2148	Micronesia: Yap State	Nov 2013
	AB860301	CHIKV-13-112A	Philippines	2013
	HM045790	PhH15483	Philippines	Jul 1985
	HM045791	JKT23574	Indonesia	1983
	HM045810	TH35	Thailand	1958
	HM045808	3412-78	Thailand	1978
ECSA	AB455494	SL10571	Sri Lanka	Dec 2006
West African	HM045786	IbH35	Nigeria	Jul 1964

*ECSA, East/Central/South African.
